# Longitudinal Association Between the Consumption of Vegetables, Fruits, and Red Meat and Diabetes Disease Burden: An Analysis of Multiple Global Datasets

**DOI:** 10.3390/nu17071256

**Published:** 2025-04-03

**Authors:** Manqiong Yuan, Juan Wang, Lifen Jin, Liangwen Zhang, Ya Fang

**Affiliations:** 1 Key Laboratory of Health Technology Assessment of Fujian Province, School of Public Health, Xiamen University, Xiamen 361102, China; yuanmanqiong@163.com (M.Y.); wangjuan09j@163.com (J.W.); jinlifen001@163.com (L.J.); lwzhang@xmu.edu.cn (L.Z.); 2 The Center for Aging and Health Research, School of Public Health, Xiamen University, Xiamen 361102, China

**Keywords:** longitudinal analysis, diabetes disease burden, dietary factors, vegetable, fruit, red meat

## Abstract

**Background:** Dietary factors, such as vegetable, fruit, and red meat consumption, have varying effects on the disease burden of diabetes, the world’s third leading health concern. This study aims to evaluate the global impact of vegetable/fruit/red meat consumption on disease burdens. **Methods:** Diabetes disease burden, vegetable/fruit/red meat consumption, and covariates data were obtained from the Global Burden of Disease Study (GBD) 2021, Food and Agriculture Organization (FAO), and WHO, respectively, and matched by country/region and year. Global vector maps assessed the status of diabetes disease burden and the consumption of three dietary factors in 2021, and their trends from 2010 to 2021 were illustrated through local regression curves. Generalized additive mixed models (GAMMs) were used to analyze relationships, with weights assigned based on log-transformed values relative to the mean population of each country. **Results:** A comprehensive dataset spanning 2010–2021, encompassing 175 countries/regions, was successfully matched and utilized in the analysis. In 2021, Oceania had the highest diabetes burden, whereas East Asia had a lower one. Globally, the disease burden increased from 2010 to 2021, accompanied by rising per capita vegetable and fruit consumption but declining red meat consumption. Vegetable consumption was inversely correlated with the age-standardized incidence rate (ASIR) and exhibited a “J-shaped” curve with the age-standardized mortality rate (ASMR) and age-standardized disability-adjusted life year (DALY) rate (ASDR) (nadir at approximately 80 kcal/capita/day (kcal/cap/day). Fruit consumption had a “U-shaped” relationship with ASDR (nadir at approximately 100 kcal/cap/day). Red meat consumption was negatively correlated to ASIR and had a “U-shaped” relationship with ASMR and ASDR (nadir at 200 kcal/cap/day). **Conclusions:** The global diabetes disease burden is heavy, and dietary consumption varies widely. Vegetable-related risks differ between diabetics and non-diabetics. Proper fruit consumption decreases ASDR. Moderate red meat increases can reduce the disease burden, but excessive consumption increases ASMR and ASDR.

## 1. Introduction

Diabetes, a metabolic disorder primarily attributed to a poor lifestyle, is caused by insufficient insulin secretion and/or impaired insulin utilization. Long-term diabetes damages tissues and organs such as the eyes, nerves, the kidneys, and cardiovascular organs, and complications jeopardize every tissue and organ in the body. Taking cardiovascular disease as an example, people with diabetes have a 2 to 3 times higher risk of developing this type of disease than their counterparts [1]. As of 2021, 537 million adults worldwide were living with diabetes, and it was expected to increase to 783 million by 2045 [2]. The “Global Compact on Diabetes” underscores the urgent need for practical measures to achieve the five 2030 targets for global diabetes coverage and treatment [3]. Overall, diabetes has become a critical public health issue that necessitates effective interventions to slow down the progression of diabetes globally.

A balanced diet plays a crucial role in managing the disease burden associated with diabetes. By utilizing the Global Burden of Disease Study (GBD) 2019 database, a study revealed that food consumption factors accounted for around 25.7% of disability-adjusted life years (DALYs) for type 2 diabetes worldwide [4]. Additionally, unhealthy food consumption indirectly exacerbates the disease burden by elevating the likelihood of being overweight or obese [5]. Among existing studies of dietary factors, within the realm of plant-based foods, whole grains, vegetables, and fruits are often associated with a significant disease burden. In the field of animal food, the consumption of red meat is in the leading position [6]. Guidelines from the WHO emphasized that a healthy diet, with the consumption of vegetables, fruits, and other diverse foods, can prevent and delay the development of diabetes [7]. While most studies indicated an inverse correlation between vegetable and fruit consumption and diabetes risk [8,9], a minority suggest no significant association, though green leafy vegetables may offer protective benefits [10]. Furthermore, there is significant controversy over the impact of red meat on the disease burden of diabetes, and there are different conclusions regarding its effects on blood glucose and insulin biomarkers [11]. These discrepancies may be intimately associated with regional differences. Thus, it is imperative to adopt a global perspective to understand the specific impact of these three dietary consumption factors on diabetes. However, existing studies mostly focus on the individual level and pay insufficient attention to the regional heterogeneity of dietary factors. The nonlinearity between dietary consumption and the disease burden of diabetes has not been fully discussed at the global level. The association between regional consumption patterns and the disease burden of diabetes requires further exploration [12].

Therefore, our research would be conducted around the following aspects: First, to comprehend the global distribution of the diabetes disease burden and the per capita consumption of vegetables, fruits, and red meat in 2021. Second, to examine the trends of the diabetes disease burden and the per capita consumption of three dietary factors within 2010–2021. Third, to determine the longitudinal correlation between the disease burden and the per capita consumption of three dietary factors via a generalized additive mixed model (GAMM). By analyzing the longitudinal association of dietary consumption with the diabetes disease burden from a global perspective, this would provide data support for the research on the diabetes disease burden and fill a gap in group-level research.

## 2. Materials and Methods

### 2.1. Data Sources

**Figure 1:**
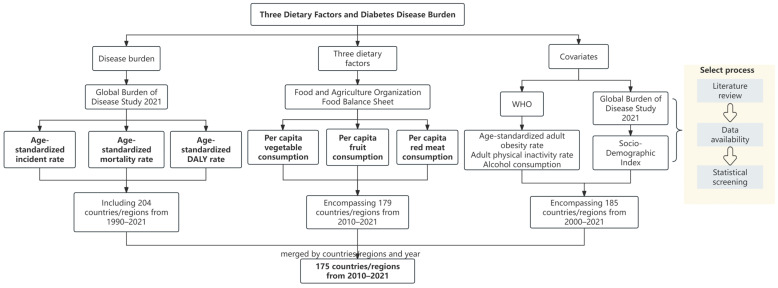
Flow chart of the selection process for the data.

In this study, the flow chart of data screening is shown in Figure 1. Country/region names were standardized according to the United Nations’ nomenclature, and missing data were addressed via interpolation or exclusion.

The data for the diabetes disease burden (age-standardized incidence/mortality/DALY rate, abbreviated as ASIR/ASMR/ASDR) from 204 countries or regions from 2010 to 2021 were extracted from the GBD 2021. The data for the GBD came from the death registration system, vital registration, verbal autopsy, and mortality monitoring [13]. Age-standardized data could eliminate the influence of factors such as age structure and population aging on the data and facilitate the comparison and analysis of data from different countries/regions.

The study incorporated data on vegetable, fruit, and red meat consumption from 2010 to 2021, derived from the Food and Agriculture Organization (FAO) Food Balance Sheet (FBS) and expressed in kcal/capita/day (kcal/cap/day). The FAO estimates the national food supply through government questionnaires, categorizing data into total supply (total supply of the country), usage (consumer-available food), and per capita supply [14]. Per capita supply was calculated by dividing the food supply by the actual consumer population data and was presented as a combination of calorie values and protein and fat content. For example, 100 g of vegetables typically provide 20–30 kcal, while a 100 g apple contains approximately 53 kcal. Red meat, depending on its fat content, ranges from 202 to 395 kcal per 100 g [15]. It is worth noting that the health effects of whole grains are mainly achieved through dietary fiber and a low glycemic index rather than energy contribution [16]. Our study focused on other dietary factors using the energy distribution index. Given that the consumption of whole grains is relatively low in the energy dimension, incorporating grains might have affected the judgment of the associations between dietary factors using this index. Therefore, due to data limitations, whole grains were not included in this study. This study only included three dietary factors (vegetables, fruits, and red meat) and regarded the per capita supply as the per capita consumption.

We selected covariates through a literature review that identified potential confounders (e.g., the overweight rate, physical inactivity rate, socio-demographic index [SDI], and smoking prevalence) associated with diabetes disease burden. The overweight rate (defined as a body mass index [BMI] of ≥25 kg/m^2^), the adult physical inactivity rate (manifested as ≤150 min of moderate-intensity physical activity per week or ≤75 min of vigorous-intensity physical activity per week, or its equivalent) and alcohol consumption (defined as total alcohol consumption per capita over the age of 15) were derived from WHO. The SDI was obtained from the GBD 2021, which is a composite indicator of developmental status closely related to health outcomes and consists of three components (per capita income, years of education, and fertility) [17]. These variables were included to control for socioeconomic disparities, metabolic risks, and lifestyle factors that may confound the diet–diabetes association.

### 2.2. Method

Three phases of analysis were conducted in this study. First, global vector maps were created to visualize the 2021 data on global diabetes disease burden and per capita vegetable, fruit, and red meat consumption. This was performed to understand the contribution to global disease burdens and the three dietary groups and to further clarify how to implement targeted control measures. Second, based on the 21 GBD regions (divided according to epidemiological homogeneity and geographic proximity) [18], scatter plots and LOESS regression-fitted curves were developed for the three dietary factors and disease burden to preliminarily explore their changes and relationships from 2010 to 2021. Finally, the GAMM was used to explore the relationship between diabetes disease burden and three dietary factors, with the log-transformed values relative to the mean population in each country/region serving as weights to mitigate the influence of substantial variations in population size across countries on the study outcomes. The GAMM is a combination of generalized additive modeling (GAM) and mixed modeling. Within this framework, it was suitable for nonlinear relationship analysis though a smoothed curve-fitting term, and the correlation of time and repeated measures was controlled by random effects [19].

Based on this, we constructed the following model:

$$Y_{i}\sim \sum_{j=1}^{p}s_{j}(X_{ij})+\beta_{i}Z_{i}+\varepsilon_{i}$$

where i refers the ith country/region; $Y_{i}$ is the diabetes disease burden, including ASIR, ASMR, and ASDR; $s_{j}(X_{ij})$ are smooth, nonlinear functions, which refers to the independent variables (including per capita vegetable, fruit, and red meat consumption) and covariates (including the SDI, overweight rate, physical inactivity rate, alcohol consumption, and year) on disease burden; p is the number of smooth terms; β is the direction and magnitude of the impact of heterogeneity on disease burden indicator Y; $Z_{i}$ refers to the random effects for the ith country/region in the model; and ε is the random error term for the ith country/region.

A GAMM analysis was performed on data from 2010 to 2021 to determine the association between the disease burden of diabetes and the per capita consumption of vegetables, fruits, and red meat. In the analysis process, the smoothing parameters were estimated using the restricted maximum likelihood (REML) method, and the model was selected through the Akaike information criterion (AIC), Bayesian information criterion (BIC), and adjusted R-squared. Covariates such as the SDI, overweight rate, physical inactivity rate, and alcohol consumption were also controlled. In order to evaluate the robustness of population-weighted models, sensitivity tests were performed for three weighting methods including unweighted, the log population, and the log-transformed population relative to the mean population.

All data analyses were performed using the open-source software R (version 4.3.2). *p*-values less than 0.05 were taken to be statistically significant.

## 3. Results

### 3.1. Geographic Distribution of Global Diabetes Disease Burden and Consumption of Vegetables, Fruits, and Red Meat

**Figure 2:**
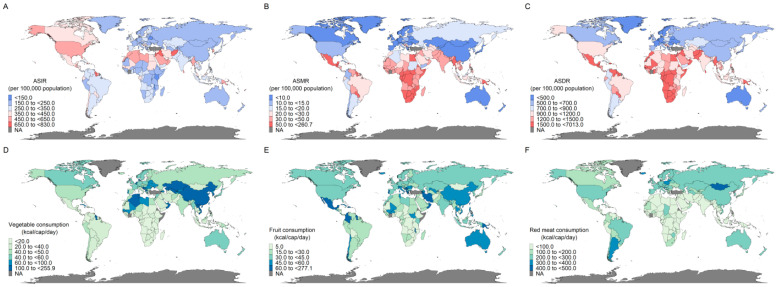
The ASIR, ASMR, and ASDR of diabetes in 2021 (**A**–**C**); the per capita consumption of vegetables, fruits, red meat in 2021 (**D**–**F**). NA represents no available data.

A comprehensive dataset spanning from 2010 to 2021, encompassing 175 countries/regions, was analyzed to assess the geographic disparities in diabetes disease burden and the consumption of three foods. In 2021, the maximum and minimum of the ASIR for diabetes were 884.21 and 135.51 per 100,000 population, corresponding to Belarus and the Marshall Islands, respectively. ASMR and ASDR showed even starker contrasts: Singapore had the lowest ASMR (2.02 per 100,000 population), while Fiji had the highest ASMR (266.11 per 100,000 population) and ASDR (7387.84 per 100,000 population), surpassing France’s minimal ASDR (354.54 per 100,000 population) by 20 times. The Oceania region (e.g., Fiji and the Marshall Islands) had the highest disease burden across all three metrics, whereas Eastern Sub-Saharan Africa, the Asia–Pacific High-Income region, and Australasia showed the lowest ASIR, ASMR, and ASDR, respectively. Of note, Sub-Saharan Africa had the seventh highest ASIR but the second highest ASMR and ASDR (Table S1 and Figure 2A–C).

Dietary consumption patterns mirrored these geographic divides. The highest per capita vegetable consumption was observed in East Asia (i.e., China and North Korea), with an average consumption of 255.63 kal/cap/day. The Caribbean had the highest fruit consumption, followed by Oceania (i.e., Australia and New Zealand), both higher than 100 kcal/cap/day. Southern Latin America consumed the most red meat (328.48 kcal/cap/day), contrasting sharply with Sub-Saharan Africa’s lower consumption across the three foods (Figure 2D–F).

### 3.2. Changes in Diabetes Disease Burden and per Capita Vegetable, Fruit, Red Meat Consumption

From 2010 to 2021, global diabetes disease burden increased steadily, with ASIR and ASDR rising by 20.8% and 14.0%, respectively. All 21 GBD regions experienced ASIR growth, ranging from 8.3% to 36.7%, especially in Southern Latin America, which had the highest ASIR. Also, trends in ASMR and ASDR across 21 GBD regions varied, and we grouped them into four distinct patterns: (1) an increase in both ASMR and ASDR (e.g., South Asia, with a 1266.8% ASMR and Oceania, with a 384.2% ASDR); (2) a decrease in both ASMR and ASDR (e.g., Western Saharan Africa, with an −86.2% ASMR and North Africa and Middle East, with an −86.5% ASDR); (3) an increase in ASMR but a decrease in ASDR (e.g., Caribbean and East Asia); and (4) a decrease in ASMR but an increase in ASDR (e.g., Andean Latin America and Central Asia) (Table 1).

Concurrently, there was a consistent and significant rise in global per capita consumption of vegetables and fruits, while red meat exhibited a declining trend. Furthermore, the per capita consumption of three dietary factors also varied significantly across different regions. Some regions (e.g., Central Asia and East Asia) showed an upward trend in the changes in all the three dietary factors’ consumption. Notably, Central Asia saw the steepest increases in vegetables (54.5%) and red meat (22.3%), whereas Australasia recorded the largest reduction in red meat (−17.3%). Approximately 25% of regions (e.g., Central Latin America and Eastern Europe) experienced a decrease in per capita vegetable consumption alongside increases in both fruit and red meat consumption, while the consumption trends for the three dietary factors of other regions (e.g., Caribbean and Southeast Asia) were similar to global (Table S2).

### 3.3. Diabetes Disease Burden to per Capita Vegetable, Fruit, Red Meat Consumption by 21 GBD Regions

Nonlinear relationships were observed between the per capita consumption of vegetables, fruits, and red meat and the disease burden of diabetes across regions (Figure S1). For vegetables, some regions (e.g., East Asia and Central Europe) revealed a high correlation with the model-predicted trend, some (e.g., Eastern Europe and Eastern Sub-Saharan Africa) with significantly lower ratios than model-predicted values, whereas Oceania showed a nearly three times higher ratio than expected. Fruit consumption also demonstrated mixed associations. The Sub-Saharan region aligned closely with the predictions, while Oceania and Central Latin America showed a heavier burden than expected, contrasting with high-income Asia Pacific’s and Western Europe’s lower-than-predicted values. Red meat consumption was more evenly distributed around the fitted curve across regions, except the higher burden in Oceania and lower burden in regions such as Eastern Europe and Southeast Asia.

### 3.4. GAMM Analyzes the Trend Between Diabetes Disease Burden and Per Capita Vegetable, Fruit, and Red Meat Consumption

**Figure 3:**
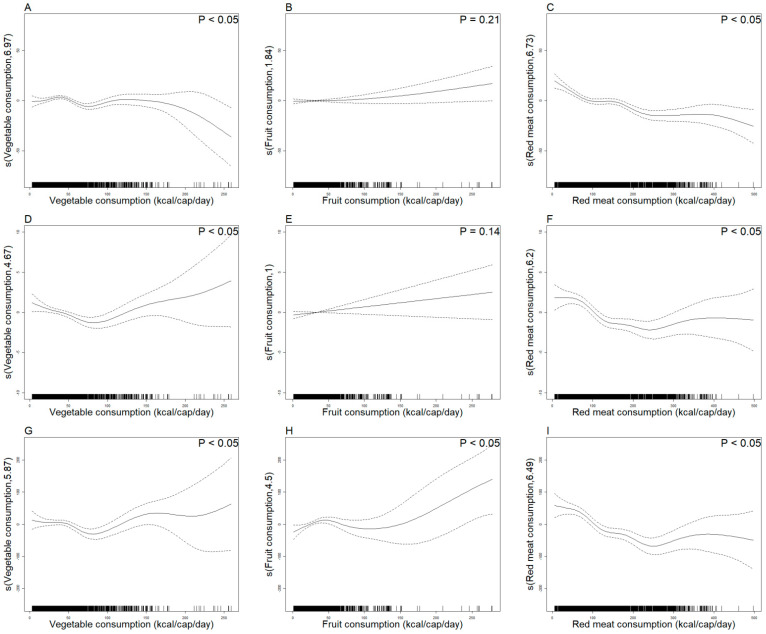
Per capita consumption of vegetables, fruits, and red meat in relation to ASIR (**A**–**C**), ASMR (**D**–**F**), and ASDR (**G**–**I**) based on GAMM, respectively. S () for vertical coordinate represents spline smoothing, where the number 1 indicates linearity, and larger values indicate stronger nonlinearity. Dashed lines refer to the 95% confidence interval. Solid lines refer to the relationship curve between the three dietary consumption and the disease burden.

The GAMM analysis indicated that the spline smoothing parameters of per capita vegetable, fruit, and red meat consumption and diabetes disease burden were all greater than one, with evidence of a nonlinear relationship. Based on the model inflection point analysis, vegetable consumption exhibited a “J-shaped” relationship with ASMR and ASDR. However, when the consumption was low (150 kcal/cap/day), the change in ASIR was relatively flat. When vegetable consumption was below 80 kcal/cap/day, a higher consumption reduced both metrics, but when it exceeded this threshold, the risks increased sharply (Figure 3A,D,G). The relationship between per capita fruit consumption and ASIR/ASMR was not significant, whereas its association with ASDR exhibited a “U-shape” relationship. It showed rising at low consumption (<40 kcal/cap/day), falling at moderate consumption (40–100 kcal/cap/day), and rebounding at excessive consumption (>100 kcal/cap/day) (Figure 3B,E,H). For red meat consumption, a “U-shaped” relationship with both ASMR and ASDR was observed, with a minimal disease burden of about 200 kcal/cap/day, while ASIR declined linearly with higher consumption (Figure 3C,F,I). In addition, the final model included in this study can achieve a good balance between simplicity and explanation (Table S3). Sensitivity analyses were conducted to explore the effects of different weighting methods on the GAMM. The results show that the corresponding effect degree of freedom maps of the three weighting methods show similar patterns, which indicates the stability of the model results under different weighting strategies (Figure S2 and Table S3).

The associations between covariates (SDI, overweight rate, physical inactivity rate, and alcohol consumption) and diabetes disease burden are presented in Figure S3. The SDI positively correlated with ASIR but showed an inverse “U-shaped” relationship with both ASMR and ASDR. Once the value of SDI was beyond 0.55, rising development levels paradoxically reduced ASMR and ASDR (Figure S3A,E,I). The overweight rate exacerbated the disease burden, underscoring its role as a primary modifiable risk factor (Figure S3B,F,J). The physical inactivity rate showed an inverse “U-shaped” relationship with ASDR beyond a prevalence of 55%, whereas it was linearly associated with increased ASIR and ASMR (Figure S3C,G,K). Alcohol consumption was positively correlated with ASIR, while the relationship with ASMR and ASDR showed an inverse “U-shaped” relationship, and the critical point was 12 L (Figure S3D,H,L).

## 4. Conclusions

This study integrated data from the GBD 2021, FAO, and WHO to assess the population-level impact of three dietary factors (vegetables, fruits, and red meat) on diabetes disease burden. Additionally, it offered macro-level evidence of dietary interventions and health policies from the population perspective.

From 2010 to 2021, diabetes disease burden continued to increase along with population aging and lifestyle changes. The burden of the disease in East Asia was generally low, which may be related to the high consumption of vegetables and fruits. Taking China’s “Jiangnan model” as an example, a large amount of vegetable and fruit in season, supplemented by the cooking method of clear soup cooking, can maximize the retention of nutritional ingredients and reduce the formation of advanced glycation end products (AGEs) [16]. At the same time, it is also inseparable from the national policy of promoting the increase in vegetable consumption [20]. Despite a moderate level of food consumption, Oceania faces a heavy disease burden. Most of these regions are Pacific islands, where the food source is mostly imported processed food, which undermines traditional eating habits, and the local population has a high genetic susceptibility. As a result, it may also face a high burden of disease [21,22]. In addition, Sub-Saharan Africa ranked seventh in ASIR among the 21 GBD regions but second in both ASMR and ASDR, suggesting potential mismatches between disease incidence and healthcare capacity. This likely stems from healthcare systems prioritizing infectious diseases over chronic conditions, thereby limiting people’s access to diabetes diagnosis and care and increasing the probability of complications and death [23].

Vegetable consumption demonstrated a continuous upward trend and was negatively correlated with the ASIR of diabetes, but the association was relatively mild when vegetable consumption was at a low level (<150 kcal/cap/day). And vegetable consumption had a “J-shaped” relationship with ASMR and ASDR. Vegetables are rich in dietary fiber, which could improve glycemic control and other risk factors for diabetes, such as cholesterol levels and body weight [10], thus reducing the disease burden of diabetes, especially in healthy people. Moderate consumption (<80 kcal/cap/day) can reduce the disease burden of diabetes through the regulation of intestinal microbiota and antioxidant [9]. Excessive consumption (>80 kcal/cap/day) may lead to insufficient consumption of essential nutrients (e.g., protein, vitamins, and minerals), nutritional imbalance, and inadequate essential amino acids. These deficiencies could accelerate the development of metabolic risk factors (e.g., obesity and dyslipidemia), thereby increasing ASMR and ASDR [24,25]. In addition, high-fat, high-temperature cooking processes may produce AGEs, and excessive consumption will promote oxidative stress and inflammation, increasing the burden of the disease [26]. This aligns with guidelines advocating for a diversified diet in some countries’ diets [27,28]. Overall, the association between vegetable consumption with disease burden is consistent with that of the EPIC-InterAct cohort, which indicated a significant reduction in disease risk when vegetable and fruit consumption reached 400 g/cap/day. However, there are differences in the appropriate amount thresholds we identified for vegetables (80 kcal/cap/day ≈ 320 g/cap/day), which may be related to differences in calorie density conversion [8,29].

Likewise, per capita fruit consumption was rising in most countries, but the relationships between fruit consumption and ASIR and ASMR were not significant, which was also similar to the conclusion in some previous studies [30]. There was a “U-shaped” relationship between fruit consumption and ASDR, with moderate consumption (40–100 kcal/cap/day) minimizing the risks. Polyphenolic compounds in fruit have anti-diabetic activity and synergistically improve insulin secretion through various pathways such as the inhibition of α-glucosidase [31]. A certain amount of fruit consumption had a moderate effect on blood sugar, but excessive fructose (>100 kcal/cap/day) might impair sensitivity through insulin resistance and oxidative stress [32]. PREDIMED-Plus showed an inverse linear association between fruit consumption and disease burden, which may be explained by the fact that the participants were more likely to adhere to the Mediterranean diet, which minimized the side effects of excessive fruit consumption [33]. Hence, we hold that a balanced amount of fruit consumption could fulfill the body’s nutrient consumption requirements.

Per capita red meat consumption exhibited a significant downward trend, and it was negatively correlated with ASIR but had a “U-shaped” relationship with both ASMR and ASDR. Moderate consumption of red meat can ensure that heme iron maintains normal glucose metabolism. At the same time, moderate consumption of high-protein lean meat can reduce postprandial blood glucose, insulin, and triglyceride values [29,34,35]. However, excessive consumption, especially the high content of saturated fat, reduces β-cell function and insulin sensitivity while increasing the body mass index and increasing the risk of diabetes [36]. Red meat consumption was below the recommended consumption of 128 kcal/cap/day in many regions of the world [37]. Similarly, the association between red meat consumption and disease burden is controversial with existing studies. Our “U-shaped” curve suggests a protective window for moderate consumption of red meat, which may be related to the fact that these studies were mostly limited to a certain region and a certain point in time [36]. For these three dietary factors, there was a large difference in the range of moderate consumption, which may be related to the glycemic index (GI) of different types of food, thus producing different energy differences. In these regions, appropriate red meat consumption and dietary modification could effectively reduce the disease burden of diabetes. On the contrary, for some regions, like the United States, Europe and other regions, red meat consumption stayed at a high level, emphasizing the importance of reducing red meat consumption [38,39].

Covariates (SDI, overweight rate, physical inactivity rate, and alcohol consumption) analysis revealed socioeconomic and behavioral drivers. Interestingly, SDI had a significant positive relationship with ASIR, but an inverted “U-shaped” relationship with both ASMR and ASDR. Regions with higher SDI levels (e.g., the United States and Canada) tended to have higher ASIR but lower ASMR and ASDR. In regions (e.g., sub-Saharan Africa) where SDI levels were lower, the disease burden was reversed. With the gradual improvement of SDI levels in a country/region, the popularization of basic medical facilities and the popularization of the concept of health education would gradually reduce the ASDR and ASMR of diabetes [40]. Simultaneously, more exposure to bad lifestyle habits would become a new risk factor, raising ASIR. In contrast, low SDI countries tended to spend more on communicable diseases and less on non-communicable diseases including diabetes [41]. The overweight rate and physical inactivity rate were positively correlated with the diabetes disease burden, which was consistent with trends in previous studies [42,43,44]. Adequate physical activity can reduce the risk of obesity while reducing the risk of diabetes in a dose-dependent manner [44]. Maintaining a certain amount of alcohol consumption for a long time may lead to decreased insulin sensitivity, enhance insulin resistance, and increase the risk of ASIR. Moderate alcohol consumption can increase the level of high-density lipoprotein cholesterol and reduce the risk of ASMR and ASDR. However, as consumption increases, the threshold of metabolism is exceeded, resulting in accelerated blood glucose fluctuations. However, those who are dependent on heavy alcohol consumption tend to develop the disease faster, which is manifested as the “false decline” of ASMR and ASDR [45,46]. Therefore, when improving SDI levels, countries/regions should focus on increasing the number of fitness and exercise places and actively encouraging people to participate in physical activities. For individuals, it is also important to increase physical activity, regularly exercise, and drink in moderation to reduce the risk of obesity.

The study integrated three global publicly available data sources and fused data from different dimensions, which significantly improved the utilization and validity of the data. We explored the association between the three commonly used foods and diabetes from a population perspective, and the findings may provide instructive recommendations for different countries/regions to develop dietary guidelines. However, there are some limitations that need to be acknowledged. Firstly, the data have some limitations that should be considered. For example, FAO’s pre-2010 data absence restricts the dataset to 2010–2021, impeding long-term trend prediction. FAO data have biases like equating supply with consumption sans waste, thus accounting and underestimating low-density food (e.g., grains) calorie intake. Additionally, GBD’s disease burden estimates (relying on other covariates) and WHO self-report data may be underestimated. Secondly, although a number of covariates have been included in our study, there are some other confounding factors measured (e.g., dietary factors, cooking methods, and lifestyle) that may bias the observed results. Thirdly, the FAO did not provide data by gender, and the oversimplified regional dietary proxies (e.g., ignoring cultural practices) may obscure nuanced associations.

## Figures and Tables

**Table 1:** The disease burden of diabetes ^a^ (per 100,000 population) in 2010 and 2021 and the relative value of changes for 21 GBD regions.

Region	2010			2021			Relative Value of Change (%) ^b^			Pattern
	ASIR	ASMR	ASDR	ASIR	ASMR	ASDR	ASIR	ASMR	ASDR	
Global	237.94	19.12	803.41	287.31	19.61	916.25	20.80	2.60	14.00	\
Andean Latin America	771.21	96.42	3306.76	930.48	89.31	3433.61	20.80	−70.90	97.20	4
Australasia	341.80	20.04	885.57	429.95	16.16	969.10	15.00	−24.70	−46.90	2
Caribbean	8613.66	1171.72	41,557.15	9840.88	1055.52	42,079.17	22.70	286.60	−11.40	3
Central Asia	1879.00	179.84	7939.18	2350.52	156.66	8163.55	13.20	−64.50	32.70	4
Central Europe	3347.95	207.59	9731.14	3719.62	213.79	10,401.60	13.70	−25.90	60.40	4
Central Latin America	2900.18	302.04	12,207.56	3331.36	337.78	14,115.18	21.10	86.80	7.50	1
Central Sub-Saharan Africa	1619.17	352.13	10,678.62	1894.98	366.75	11,557.65	24.70	−81.20	10.20	4
East Asia	657.71	56.17	2271.89	782.99	48.07	2354.17	28.00	50.00	−6.60	3
Eastern Europe	1105.74	31.25	2704.18	1360.24	68.57	4008.63	18.70	79.50	−5.00	3
Eastern Sub-Saharan Africa	2452.37	688.16	19,027.46	2807.69	686.88	19,575.82	25.70	293.90	113.90	1
High-income Asia Pacific	1427.55	95.14	4150.01	1815.18	67.30	4298.09	19.50	−69.50	116.60	4
High-income North America	720.94	37.90	1796.89	1031.02	30.16	2120.67	33.50	76.00	−41.60	3
North Africa and Middle East	8949.61	892.83	31,445.52	11,193.22	801.01	33,656.39	21.10	−77.30	−86.50	2
Oceania	10,355.97	2109.40	67,391.16	11,905.06	2101.08	70,709.09	26.00	120.00	384.20	1
South Asia	1226.03	167.78	5464.40	1477.91	178.23	6160.72	8.30	1266.80	3.00	1
Southeast Asia	4134.20	504.79	17,626.73	5148.67	493.28	19,133.57	20.90	−15.10	13.20	4
Southern Latin America	749.43	52.72	2124.25	955.58	44.02	2260.86	36.70	−24.50	48.50	4
Southern Sub-Saharan Africa	1613.00	556.98	14,463.18	1896.95	493.35	13,606.41	19.10	−4.40	−16.70	2
Tropical Latin America	564.35	87.10	2833.34	647.93	83.53	2923.87	17.80	122.40	−27.90	3
Western Europe	4876.75	265.85	11,773.05	6014.55	206.43	12,554.87	13.60	239.80	131.70	1
Western Sub-Saharan Africa	4367.65	731.09	23,206.38	5154.96	745.13	24,987.03	13.40	−86.20	−17.20	4

^a^: Age-standardized DALY rate: ASDR; age-standardized incident rate: ASIR; age-standardized mortality rate: ASMR. ^b^: Relative value of change = (Disease burden in 2021-Disease burden in 2010)/Disease burden in 2010.

## Data Availability

The datasets generated and analyzed during the current study are available in the GBD 2021 at https://vizhub.healthdata.org/gbd-results/ (accessed on 8 October 2024), FAO at https://www.fao.org/faostat/zh/#data/FBS (accessed on 8 October 2024), and WHO at https://www.who.int/data/gho/data/indicators (accessed on 8 October 2024).

## Supplementary Materials

The following supporting information can be downloaded at https://www.mdpi.com/article/10.3390/nu17071256/s1, Figure S1. ASIR, ASMR, and ASDR of diabetes (2010–2021) in 21 GBD regions attributed to the per capita consumption of vegetables (A–C), fruits (D–F), and red meat (G–I). The solid black line represents the expected value based on the per capita consumption of vegetables, fruits, and red meat, respectively. ASIR: age-standardized incident rate; ASMR: age-standardized mortality rate; ASDR: age-standardized DALY rate; Figure S2. The SDI, overweight rate, physical inactivity rate, and alcohol consumption in relation to ASIR (A–D), ASMR (E–H), and ASDR (I–L) based on GAMM, respectively. S () for vertical coordinate represents spline smoothing, where the number 1 indicates linearity, and larger values indicate stronger nonlinearity. ASIR: age-standardized incident rate; ASMR: age-standardized mortality rate; ASDR: age-standardized DALY rate; Figure S3. Sensitivity analysis of the GAMM fitting results for different weighting methods; Table S1. The 21 GBD regions corresponding to each country/region in the world; Table S2. The per capita consumption (kcal/cap/day) of vegetables, fruits, and red meat in 2010 and 2021 and the relative value of changes by 21 GBD regions; Table S3. Model selection of key indicators and sensitivity analysis of GAMM fitting results under diverse weighting methods.

## Abbreviations

The following abbreviations are used in this manuscript:

AGEs	Advanced glycation end products
AIC	Akaike information criterion
ASDR	Age-standardized DALY rate
ASIR	Age-standardized incident rate
ASMR	Age-standardized mortality rate
BIC	Bayesian information criterion
BMI	Body mass index
DALY	Disability-adjusted life years
FAO	Food and Agriculture Organization
FBS	Food balance sheet
GAM	Generalized additive modeling
GAMM	Generalized additive mixed model
GBD	Global Burden of Disease Study
GI	Glycemic index
REML	Restricted maximum likelihood
SDI	Socio-demographic index
