# Supplementary material for: Longitudinal Association Between the Consumption of Vegetables, Fruits, and Red Meat and Diabetes Disease Burden: An Analysis of Multiple Global Datasets
Source: Nutrients. 2025 Apr 3;17(7):1256. doi: 10.3390/nu17071256 (PMC11990858; doi:10.3390/nu17071256)
Supplement: Supplementary file 1 [file nutrients-17-01256-s001.zip › nutrients-3523645-supplementary.pdf]

# Supplemental Materials

## Supplementary Figures

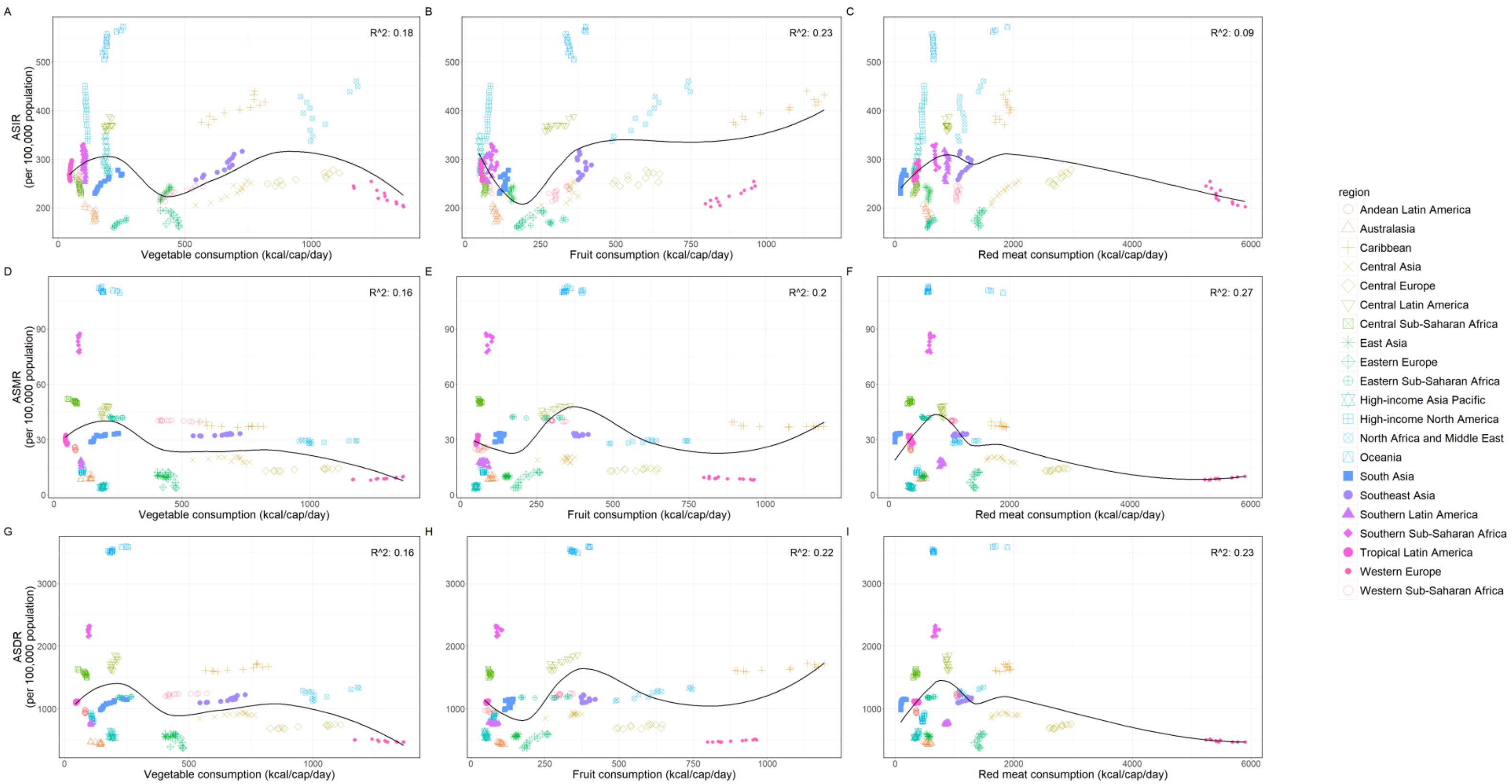


**Figure S1** ASIR, ASMR, and ASDR of diabetes (2010-2021) in 21 GBD regions attributed to per capita consumption of vegetable (*A-C*), fruit (*D-F*), and red meat (*G-I*). The solid black line represents the expected value based on the per capita consumption of vegetable, fruit, and red meat, respectively. ASIR: age-standardized incident rate; ASMR: age-standardized mortality rate; ASDR: age-standardized DALY rate.

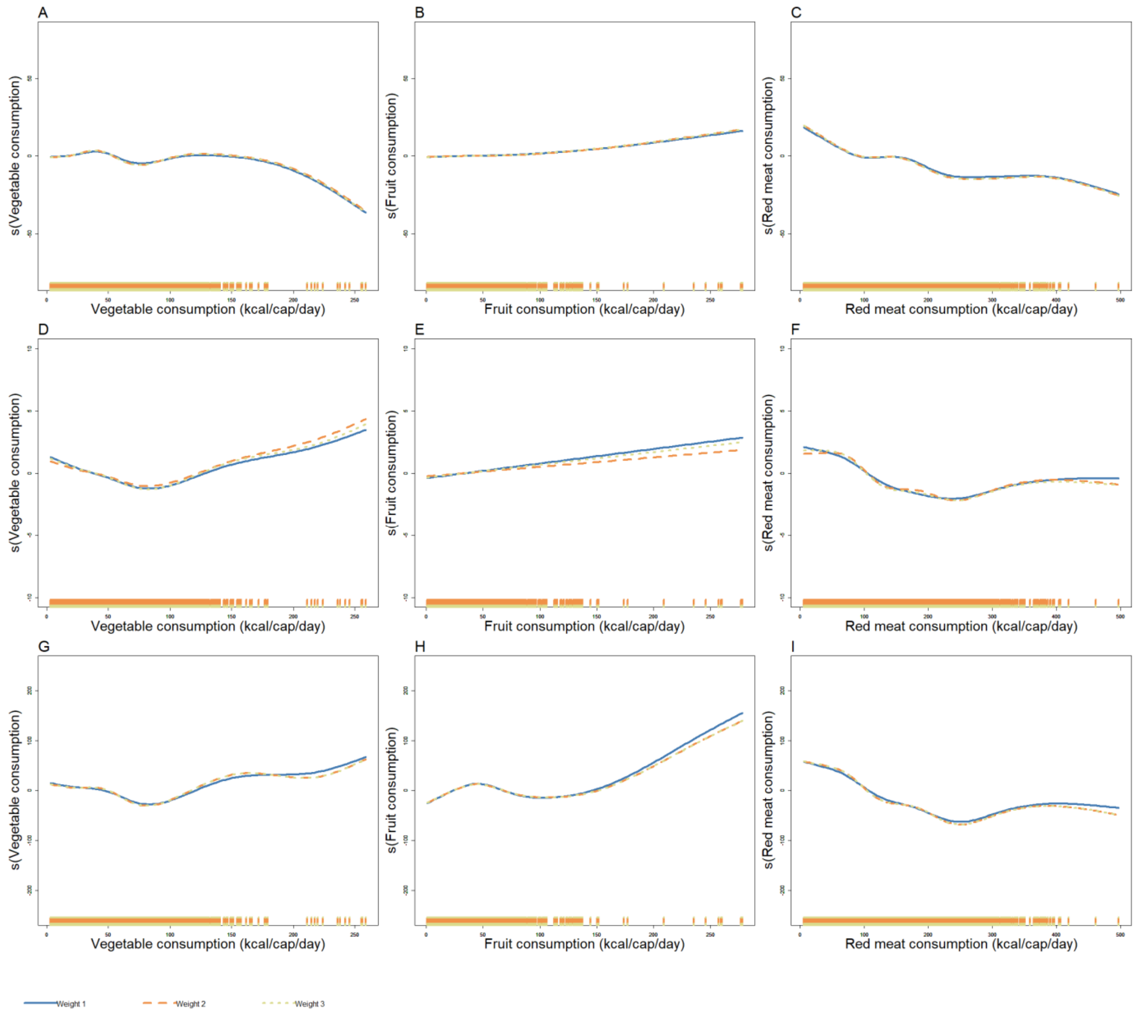


**Figure S2** Sensitivity analysis of the GAMM fitting results for different weighting methods.

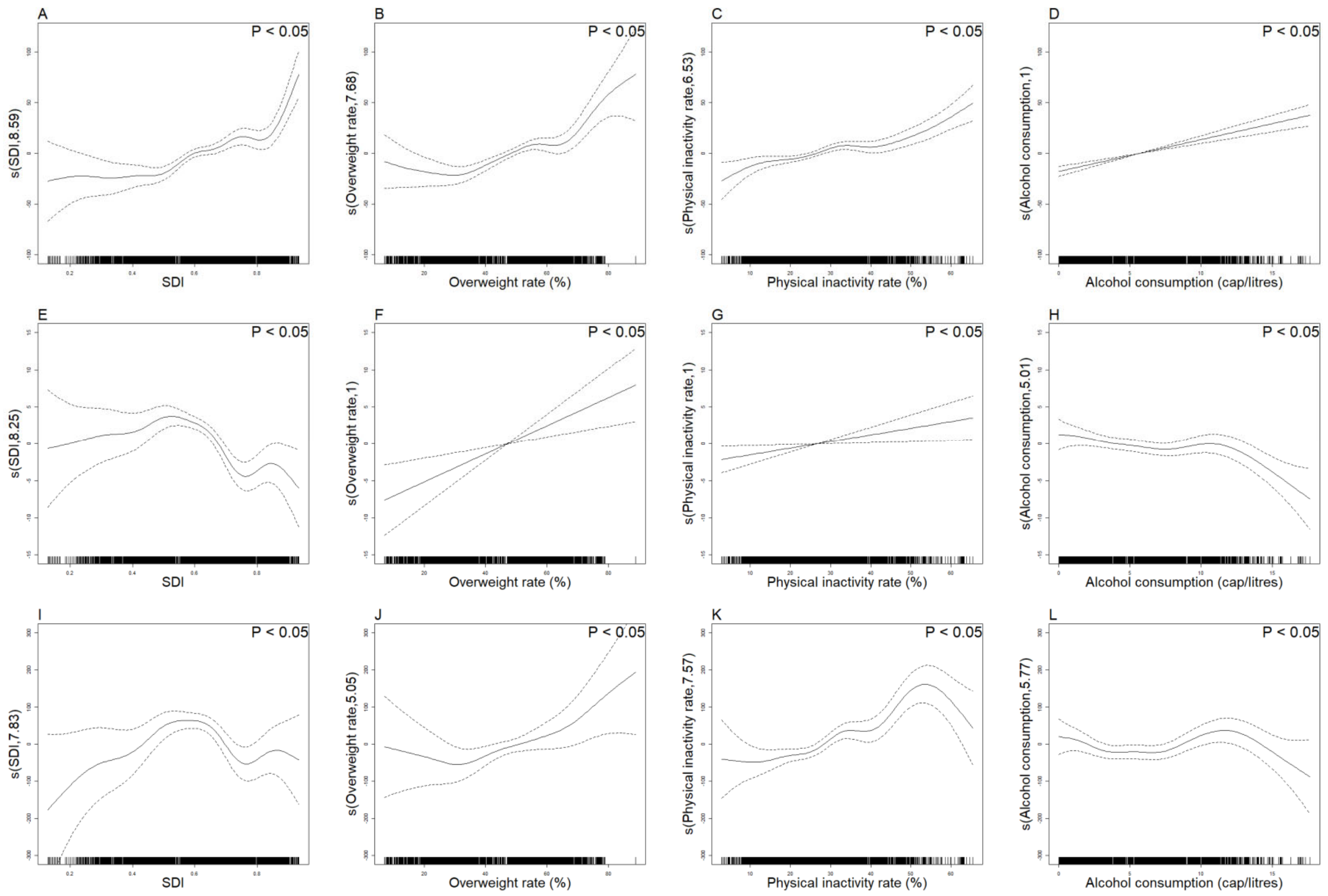


**Figure S3** SDI, overweight rate, physical inactivity rate and alcohol consumption in relation to ASIR (*A-D*), ASMR (E-*H*), and ASDR (*I-L*) based on GAMM, respectively. s () for vertical coordinate represents spline smoothing, where the number 1 indicates linearity, and larger values indicate stronger nonlinearity. ASIR: age-standardized incident rate; ASMR: age-standardized mortality rate; ASDR: age-standardized DALY rate.

## Supplementary Table

**Table S1** 21 GBD regions corresponding to each country/region in the world

| GBD regions | Countries/ regions |
|---|---|
| Andean Latin America | Bolivia (Plurinational State of), Ecuador, Peru |
| Australasia | Australia, New Zealand |
| Caribbean | Antigua and Barbuda, Bahamas, Barbados, Belize, Bermuda, Cuba, Dominica, Dominican Republic, Grenada, Guyana, Haiti, Jamaica, Puerto Rico, Saint Kitts and Nevis, Saint Lucia, Saint Vincent and the Grenadines, Suriname, Trinidad and Tobago, United States Virgin Islands |
| Central Asia | Kazakhstan, Kyrgyzstan, Mongolia, Tajikistan, Turkmenistan, Uzbekistan |
| Central Europe | Montenegro, North Macedonia, Poland, Romania, Serbia, Slovakia, Slovenia |
| Central Latin America | Nicaragua, Panama, Venezuela (Bolivarian Republic of) |
| Central Sub-Saharan Africa | Angola, Central African Republic, Congo, Democratic Republic of the Congo, Equatorial Guinea, Gabon |
| East Asia | China, Democratic People's Republic of Korea, Taiwan (province of China) |
| Eastern Europe | Belarus, Estonia, Latvia, Lithuania, Republic of Moldova, Russian Federation, Ukraine, |
| Eastern Sub-Saharan Africa | Burundi, Comoros, Djibouti, Eritrea, Ethiopia, Kenya, Madagascar, Malawi, Mozambique, Rwanda, Somalia, South Sudan, Uganda, United Republic of Tanzania, Zambia |
| High-income Asia Pacific | Japan, Republic of Korea, Singapore |
| High-income North America | Canada, Greenland, United States of America |
| North Africa and Middle East | Afghanistan, Algeria, Bahrain, Egypt, Iran (Islamic Republic of), Iraq, Jordan, Kuwait, Lebanon, Libya, Morocco, Oman, Palestine, Qatar, Saudi Arabia, Sudan, Syrian Arab Republic, Tunisia, Turkey, United Arab Emirates, Yemen |
| Oceania | American Samoa, Cook Islands, Fiji, Guam, Kiribati, Marshall Islands, Micronesia (Federated States of), Nauru, Niue, Northern Mariana Islands, Palau, Papua New Guinea, Samoa, Solomon Islands, Tokelau, Tonga, Tuvalu, Vanuatu |
| South Asia | Bangladesh, Bhutan, India, Nepal, Pakistan |

| GBD regions | Countries/ regions |
|---|---|
| Southeast Asia | Cambodia, Indonesia, Lao People's Democratic Republic, Malaysia, Maldives, Mauritius, Myanmar, Philippines, Seychelles, Sri Lanka, Thailand, Timor-Leste, Viet Nam |
| Southern Latin America | Argentina, Chile, Uruguay |
| Southern Sub-Saharan Africa | Botswana, Eswatini, Lesotho, Namibia, South Africa, Zimbabwe |
| Tropical Latin America | Brazil, Paraguay |
| Western Europe | Andorra, Austria, Belgium, Cyprus, Denmark, Finland, France, Germany, Greece, Iceland, Ireland, Israel, Italy, Luxembourg, Malta, Monaco, Netherlands, Norway, Portugal, San Marino, Spain, Sweden, Switzerland, United Kingdom |
| Western Sub-Saharan Africa | Benin, Burkina Faso, Cabo Verde, Cameroon, Chad, Cote d'Ivoire, Gambia, Ghana, Guinea, Guinea-Bissau, Liberia, Mali, Mauritania, Niger, Nigeria, Sao Tome and Principe, Senegal, Sierra Leone, Togo |

**Table S2** Per capita consumption (kcal/cap/day) of vegetable, fruit, red meat in 2010 and 2021 and relative value of changes by 21 GBD regions

| Region | 2010 | | | 2021 | | | Relative value of change(%)[a] | | |
|---|---|---|---|---|---|---|---|---|---|
| | Vegetable | Fruit | Red meat | Vegetable | Fruit | Red meat | Vegetable | Fruit | Red meat |
| Global | 75.18 | 30.70 | 138.63 | 83.83 | 36.67 | 135.06 | 11.50 | 19.40 | -2.60 |
| Andean Latin America | 31.48 | 32.09 | 95.34 | 33.53 | 20.44 | 96.06 | 6.50 | -36.30 | 0.80 |
| Australasia | 59.74 | 43.95 | 298.79 | 53.23 | 46.38 | 247.25 | -10.90 | 5.50 | -17.30 |
| Caribbean | 28.50 | 70.32 | 94.59 | 42.58 | 104.41 | 89.85 | 49.40 | 48.50 | -5.00 |
| Central Asia | 63.25 | 29.24 | 166.48 | 97.74 | 41.00 | 203.63 | 54.50 | 40.20 | 22.30 |
| Central Europe | 62.74 | 34.74 | 231.87 | 66.55 | 43.78 | 254.12 | 6.10 | 26.00 | 9.60 |
| Central Latin America | 24.65 | 42.35 | 139.49 | 24.02 | 59.78 | 149.70 | -2.60 | 41.20 | 7.30 |
| Central Sub-Saharan Africa | 9.66 | 10.27 | 29.03 | 6.52 | 9.86 | 29.98 | -32.60 | -4.00 | 3.30 |
| East Asia | 209.18 | 39.46 | 276.27 | 255.63 | 52.01 | 282.87 | 22.20 | 31.80 | 2.40 |
| Eastern Europe | 57.78 | 29.54 | 184.27 | 53.99 | 31.95 | 190.21 | -6.60 | 8.20 | 3.20 |
| Eastern Sub-Saharan Africa | 16.27 | 13.24 | 41.31 | 15.92 | 18.42 | 41.31 | -2.20 | 39.20 | 0.00 |
| High-income Asia Pacific | 80.15 | 22.06 | 140.42 | 75.73 | 20.76 | 165.70 | -5.50 | -5.90 | 18.00 |
| High-income North America | 50.16 | 28.76 | 249.54 | 47.09 | 39.93 | 281.46 | -6.10 | 38.80 | 12.80 |
| North Africa and Middle East | 57.22 | 25.33 | 45.86 | 49.88 | 34.22 | 45.74 | -12.80 | 35.10 | -0.30 |
| Oceania | 46.46 | 110.34 | 83.58 | 44.19 | 102.31 | 74.61 | -4.90 | -7.30 | -10.70 |
| South Asia | 34.27 | 25.66 | 14.39 | 43.63 | 33.11 | 15.86 | 27.30 | 29.00 | 10.20 |
| Southeast Asia | 39.88 | 38.11 | 80.74 | 51.53 | 39.68 | 76.41 | 29.20 | 4.10 | -5.40 |
| Southern Latin America | 35.27 | 23.07 | 315.68 | 33.93 | 28.10 | 328.48 | -3.80 | 21.80 | 4.10 |

| | | | | | | | | | |
|---|---|---|---|---|---|---|---|---|---|
| Southern Sub-Saharan Africa | 21.39 | 9.10 | 162.20 | 17.24 | 7.83 | 161.15 | -19.40 | -14.00 | -0.60 |
| Tropical Latin America | 20.87 | 31.07 | 223.51 | 20.38 | 25.97 | 238.70 | -2.40 | -16.40 | 6.80 |
| Western Europe | 59.68 | 38.52 | 286.67 | 55.06 | 42.84 | 252.75 | -7.70 | 11.20 | -11.80 |
| Western Sub-Saharan Africa | 29.52 | 16.25 | 42.59 | 33.56 | 17.43 | 41.69 | 13.70 | 7.30 | -2.10 |

[a]: Relative value of change = (food consumption in 2021 - food consumption in 2010) / food consumption in 2010

**Table S3** Model selection of key indicators and sensitivity analysis of GAMM fitting results under diverse weighting methods

| | ASIR [a] | | | ASMR | | | ASDR | | |
|---|---|---|---|---|---|---|---|---|---|
| | **AIC** | **BIC** | **Adjusted R-squared** | **AIC** | **BIC** | **Adjusted R-squared** | **AIC** | **BIC** | **Adjusted R-squared** |
| **Type of model [b]** | | | | | | | | | |
| Based model | 17185.658 | 17247.747 | 0.994 | 10962.222 | 11024.310 | 0.994 | 24014.260 | 24076.348 | 0.996 |
| Full covariate model | 15385.151 | 15524.270 | 0.995 | 9600.307 | 9739.426 | 0.996 | 21871.164 | 22010.283 | 0.997 |
| Final model | 16432.690 | 16539.427 | 0.995 | 10633.985 | 10740.723 | 0.995 | 23342.072 | 23448.810 | 0.997 |
| **Weight method [c]** | | | | | | | | | |
| Method 1 | 16284.484 | 16391.062 | 0.995 | 10681.941 | 10788.518 | 0.995 | 23265.478 | 23372.056 | 0.997 |
| Method 2 | 16304.034 | 16410.612 | 0.995 | 10550.534 | 10657.112 | 0.995 | 23158.184 | 23264.762 | 0.997 |
| Method 3 | 16303.885 | 16410.463 | 0.995 | 10555.544 | 10662.122 | 0.995 | 23158.184 | 23264.762 | 0.997 |

[a]: ASIR: age-standardized incident rate; ASMR: age-standardized mortality rate; ASDR: age-standardized DALY rate

[b] :Based model: including three dietary factors; Full covariate model: including three dietary factors and seven covariates (SDI, overweight rate, physical inactivity rate, and alcohol consumption, greening rate, sex ratio, unemployment rate); Final model: including three dietary factors and four covariates (SDI, overweight rate, physical inactivity rate, and alcohol consumption).

[c]: Method 1=unweighted, Method 2 = log (population), Method 3= log (population)/mean [log (population)].
